# Comparative Functional Study of Thioester-containing Related Proteins in the Recently Sequenced Genome of *Biomphalaria glabrata*

**Published:** 2018

**Authors:** Mofolusho O. FALADE, Benson OTARIGHO

**Affiliations:** 1. Cellular Parasitology Programme, Cell Biology and Genetics Unit, Department of Zoology, University of Ibadan, Ibadan, Nigeria; 2. Dept. of Biological Science, Edo University, Iyamho, Edo State, Nigeria; 3. Dept. of Molecular Microbiology and Immunology, School of Medicine, Oregon Health and Science University, Portland, OR, USA

**Keywords:** *Biomphalaria glabrata*, Thioester-containing proteins, Schistosomiasis

## Abstract

**Background:**

There is paucity of information on functional relationship and characterization of *Biomphalaria glabrata* thioester-containing proteins (*Bg*TEP) to other well-annotated homologues. We performed functional characterization studies of *Bg*TEP to homologues in *Anopheles gambiae* and in disparate invertebrates.

**Methods:**

Genomic sequences of TEPs were retrieved after annotation of the *B. glabrata* genome. In addition, TEP sequences deposited in NCBI protein database were also retrieved and utilized for sequence analysis. *Bg*TEP relatedness to its other homologues as well as functional domain and protein-protein interaction analysis was performed.

**Results:**

Our analysis resulted in the identification of TEPs in a number of organisms including, *B. glabrata*, *A. gambiae*, and *Chlamys farreri.* In addition, we identified 19 TEP sequences spread across 10 animal species*.* The *B. glabrata* genome contains 14190 unannotated proteins after filtration with about 85% of its proteome annotated. The phylogenetics, functional domain and protein-protein interaction analyses suggest an immunological role for *Bg*TEP in *B. glabrata*.

**Conclusion:**

The predicted role of thioester-containing proteins to be involved in immunological role in *B. glabrata* may have a strong effect on resistance to infection.

## Introduction

Schistosomiasis is the most widespread trematode infection with an estimate of 200 million people infected in the developing world (1–6). Consequently, schistosomiasis has been reported to kill more than 200000 people per year (7, 8). The control of this disease depends primarily on treatment of infected persons with Praziquantel (9, 10) with no recommended alternative chemotherapeutic options available (11, 12) or a licensed vaccine (11, 13). A safe and among the recommended alternatives for schistosomiasis control is the targeting of the snail vector (14–17). *Biomphalaria glabrata* is the most important host in the transmission of human schistosomiasis in the Caribbean and South America. Hence, recent molecular and immunological work has focused on *Biomphalaria glabrata* (2). Therefore, *B. glabrata* provides a useful model organism for the study of schistosomiasis (18). The urge to unveil the complex paradox of interactions between this snail and the *Schistosoma* parasite has led to the completion of its genome sequence. Exploring for genes that play vital roles in the snail immunity that determine the success or failure of an infection or parasite development is of major interest (17–19).

Some identified important immune factors are the nuclear factor kappa B (NF-κB) homologues (20–29) and biomphalysin (30). The latter is a β pore-forming toxin involved in the snail immune defense against *Schistosoma mansoni*. Besides, these immune factors can be passed from generation to generation in snails, which determine resistance to parasite infection (28–30). The recently completed *B. glabrata* snail genome, which is available on vector base (https://www.vectorbase.org/organisms/biomphalaria-glabrata) (31), is facilitating exploitation of unknown, vital and novel immunological factors that can help to unveil the complex immune system of this snail against pathogens like *Schistosoma* species (19, 18). Different studies have identified thioester-containing protein (TEP) to be present in the snail genome, which could play an immunological role in *B. glabrata* (32–34). TEP in *Anopheles gambiae* was identified and its vital role was determined in its immunity (35). However, there is no report comparing the functions of TEP in *B. glabrata* (*Bg*TEP) to that of *A. gambiae*, since such findings will give a better understanding of the role the protein may play in *Schistosoma* infection and development.

In the present study, we explored the recently completed *B. glabrata* genome sequence for the present of thioester-containing proteins and carried out comparative analysis with homologues in *A. gambiae* and a disparate number of invertebrate; predict the possible role TEP might play in defense and protection of *B. glabrata* against infection.

## Materials and Methods

### Literature search and retrieval for invertebrate thioester-containing protein

We performed a thorough manual literature search using “Thioester-containing Protein plus Mollusca” and “Thioester-containing Protein plus *B. glabrata*” separately in Oct 2016 of The National Center for Biotechnology Information (NCBI) (https://www.ncbi.nlm.nih.gov/guide/proteins/) website for invertebrates TEP proteins and the resulting protein sequences were confirmed on UniProt (http://www.uniprot.org). Protein sequence homologues of a variety of insects such as *Anopheles* mosquitoes, *Drosophila* were retrieved from NCBI. These were used, as they are recognized invertebrate organisms for which numerous studies have described key aspects of their innate immune system response to pathogens. Other Mollusca such as *Azumapecten farreri* and *Euphaedusa tau* with TEP protein been sequenced were also retrieved and included in the analyses. The different proteoforms of *B. glabrata* TEP protein, deposited by (32, 36, 37) with the accession No: ACL00841.1 and AHH81765.1 were retrieved. Partial sequences were avoided during retrieval and collection. All protein sequences were retrieved in FASTA format.

### *B. glabrata* Proteome downloads and annotations

The publicly available genomic data of *B. glabrata* (BB02) (Biomphalaria-glabrata-BB02_PEPTIDES_BglaB1.4.fa.gz; BB02 strain peptide sequences, BglaB1.4 geneset), containing more than 14,000 none annotated sequences were downloaded from VectorBase, http://www.vectorbase.org, (38) and converted to FASTA format using geneious version R8 (39). Exported FASTA files of these sequences were functionally annotated on Blast2Go version 3.3 (40–43). Sequence annotation was performed by BLAST of NCBI (National Center for Biotechnology Information) http://www.ncbi.nlm.nih.gov/ database using blastp. Algorithm, non-redundant (nr) protein database, 1.0xE3 for blast expectation value and sequences with a maximum hit of 20 sequences on the average. The Blast2Go cut-off parameters used to filter out poor quality BLAST hits for the annotation were as follows: Annotation rule cut-off = 55; E-value = 1e–6; Hit-HSP overlap = 0; and the GO weight = 5.

### Structural and Functional Analyses of Thioester-containing Protein

The structural and functional analyses of each invertebrate TEP selected for this work were subjected to various physical and chemical parameter prediction by employing a web server tool, ProtParam (44). The parameters analyzed were molecular weight, theoretical pI, amino acid composition, instability index, aliphatic index and grand average of hydropathicity (GRAVY) of each protein. The presence of signal peptides and position of each sequence were checked using Signal P web tool (45) and targetP (46). SecretomeP version 2.0 for non-classically secreted protein prediction was used in determining pathways of secretion for TEP in *B. glabrata* and other species. Prediction of transmembrane helices was done by TMHMM Server v. 2.0 and validated using CCTOP webtool (47). Further confirmation and validation of theoretical pI and molecular weight was achieved using Compute pI/Mw (48–50) and AACompIdent to validate amino acid composition. PepCalc.com was used to calculate Peptide property (50). Subcellular localization of each protein was predicted using an advanced protein subcellular localization prediction tool; WoLF PSORT. String was utilized in determining proteins that bind to TEP in *B. glabrata* and other species analyzed. Functional sites and domains of each protein were obtained from Pfam 30.0 and results were validated using SMART and NCBI Conserved Domains.

### Phylogenetic tree and Evolutionary relatedness analysis

Phylogenetic trees were constructed using MEGA version 7 software and neighbor-joining method was applied in the analysis. The trees were drawn to scale, with branch lengths measured for the number of substitutions per site. We removed 19 amino acids and all ambiguous positions were removed for each sequence pair. There were 127 positions in the final dataset. The percentage of replicate trees in which the associated taxa clustered together in the bootstrap test was 1000 replicates. The constructed phylogenetic trees were validated using Robust Phylogenetic Analysis tools (http://phylogeny.lirmm.fr/phylo_cgi/index.cgi). The tree file in Newick format was exported and visualized in FigTree software version 1.4.2 for proper annotation. We went further to estimate the pairwise distance using the same version of MEGA. Analyses were conducted using the Poisson correction model. The analysis involved 19 amino acid sequences. Comparative functional domain analysis arranged along the branch of the constructed tree using MEGA was employed in the prediction of the possible functional role of *Bg*TEP.

## Results

A comprehensive search for Thioester-containing Proteins from *B. glabrata* and a variety of invertebrate genome sequences resulted in the identification of 19 invertebrate TEP sequences spread across 10 species. After a thorough literature searched for Thioester-containing protein, this protein had been well established in insects especially the chief vectors of malaria parasite, *A. gambiae* and Mollusca, *Chlamys farreri* (Farrer's scallop)*.* Nineteen invertebrate TEP sequences were used in this study that spread across 10 species including *B. glabrata.* The *B. glabrata* proteome dataset downloaded from vectorbase consist of 14190 unannotated proteins after filtration. After annotation using Blast2Go about 85% of these protein sequences were annotated while the other 15% sequences remained unannotated. About 15 proteins out of the 19 were predicted to have signal peptides computed to be localized in the secretory pathway with a strong reliability class (RC), while the remaining 4, lack signal peptides. Ten of the selected protein sequences were secreted using the non-classical pathway. The other proteins were predicted to secrete through the classical pathways. Few of the proteins were computed to have just one TMH; *A. gambiae* (AAG00600.1), *A. sinensis* (KFB36250.1), *Drosophila mojavensis* (EDW13040.2), *D. virilis* (EDW64840.1), *D. mojavensis* (EDW12892.1) and *D. mojavensis* (EDW11960.2).

**Fig. 1:**
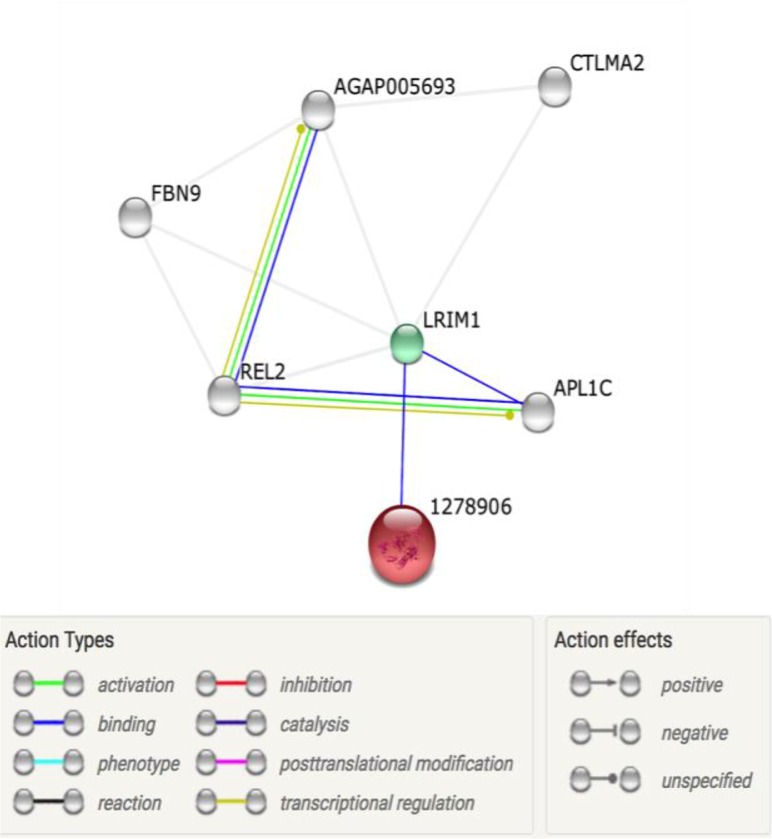
Protein-Protein interaction network of TEP in an invertebrate

Most of the protein-protein interactions prediction show that TEP binds with different proteoforms of leucine-rich immune protein (LRIM1), which in turn binds to APL1C and REL2 and other uncharacterized proteins (Fig. 1). The binding of TEP to LRIM1 is strong, judging by its affinity score of 0.845. Besides REL2 binding to AL1C, its aid in activation and translational regulation of APL1C and the uncharacterized protein. The interaction network also shows that the evidence of these bindings is both experimental determination and text mining. All interactions have a high confidence.

**Fig. 2:**
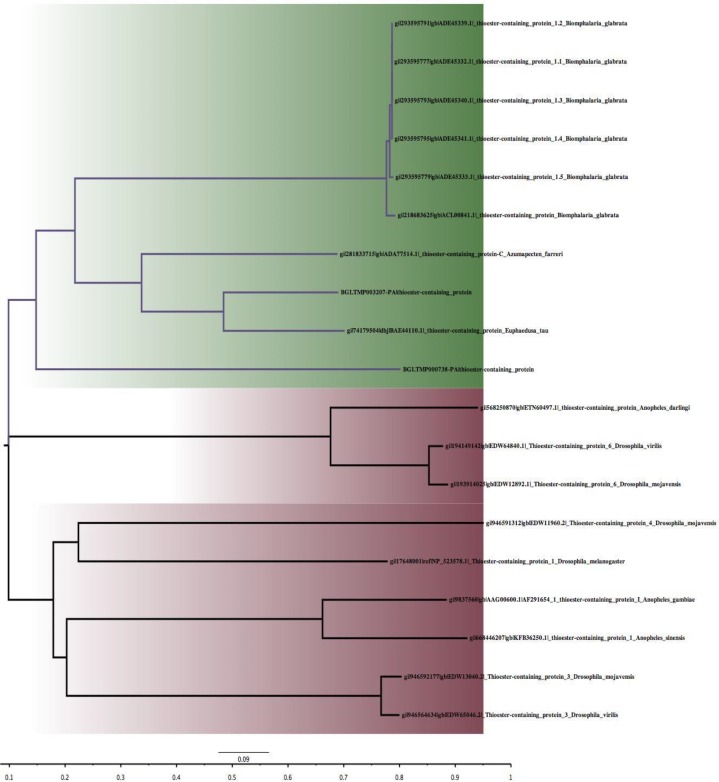
Phylogenetic analysis of the different invertebrates TEP based on neighbor-joining method. There are two main Claude, the one in green include all Mollusca species TEP, the one in brown include all Insecta species TEP

**Fig. 3:**
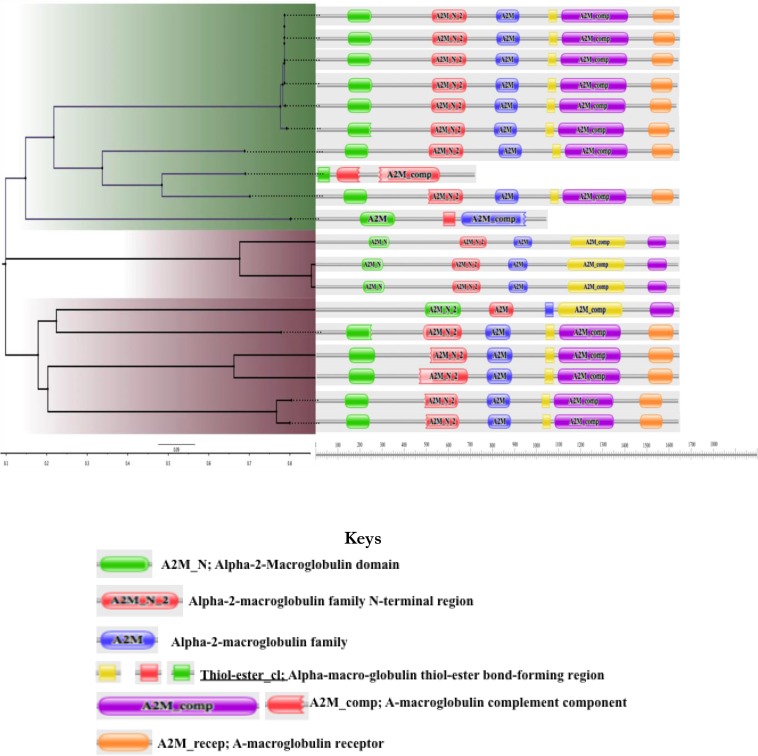
Functional domains/families of the different invertebrates TEP

The entire insect TEP selected formed a major Claude while the others from Mollusca formed another Claude (Fig. 2). Among the insect Claude, some species of *Anopheles* and *Drosophila* show similarities as seen between *A. darling* (gb|ETN60497.1) and *D. virilis* (gb|EDW64840.1) as well as *D. mojavensis* (gb|EDW12892.1). The two proteoforms of *B. glabrata* TEP shows similarity to the two other Mollusca species; *Azumapecten farreri* and *Euphaedusa tau* (Fig. 2). Functional domain analyses show that all the TEP possess similar functional motifs, which consist of different domains of alpha-2-Macroglobulin (A2M_N, A2M_N_2, A2M, A2M_comp and A2M_recep) as well as Alpha-macro-globulin thiol-ester bond-forming region (Thiol-ester_cl) Fig. 3. However, *B. glabrata* TEP identified from vectorbase dataset do not conform to this possessing just a few of the motifs. *A. darling* (gb|ETN60497.1), *D. virilis* (gb|EDW64840.1) and *D. mojavensis* (gb|EDW12892.1) lack Alpha-macro-globulin thiol-ester bond-forming regions (Fig. 3). All the functional sites matched on were obtained with high Expect (E) value.

## Discussion

The success of *Schistosoma* infection and transmission is tied to the presence of compatible intermediate snail hosts (26). Thus, proteins employed by snails in mounting defense responses to prevent infection may have a significant impact on the transmission and control of schistosomiasis (12). Consequently, exploiting snail resistance responses could limit the number of new snail infections in the wild (20, 26). Some of the proteins implicated in immune responses of snails include fibrinogen-related protein (26) and the recently identified protein, Biomphalysin (30, 27). The needs to decipher the immunity of the snail vector against *Schistosoma* infection have led to the exploitation of the recently sequenced *B. glabrata* genome to identify additional proteins useful for snail immunity (19). One of such proteins suggested to play a vital role in snail immunity is thioester-containing protein (TEP). Numerous studies have highlighted the role of TEP in a few invertebrates especially arthropods such as *Anopheles* mosquitoes and *Drosophila melanogaster* (35). Some of these works have employed molecular and computational tools in the identification and characterization of Thioester-containing Protein from other Mollusca such as from Zhikong scallop. However, none has identified the presence of thioester-containing protein in the proteome of *B. glabrata* (32, 34).

Our structural and functional analyses revealed that most of the proteins studied were stable. Some of these proteins may possibly be secreted via the non-classical pathway and others through the classical pathway. Host survival during infections is determined by the ability of the immune response proteins, some of which are secretory, interfering with parasite development (50). Our identification of TEP in *B. glabrata* may provide a probable reason to why some snails are susceptible and some resistant to schistosome infection. Identified immune molecule, fibrinogen-related protein 3 (FREP3) is important for snail defenses against schistosome infections in *Biomphalaria* (22, 25). A loss of resistance in infected snails was associated with a strong down-regulation of FREP3 and other candidate immune molecules. TEP may also function in the same way as observed in FREP3 by inducing immunity during infection. An understanding of how the susceptibility of snails in schistosomiasis endemic areas can be manipulated may result in snails with reduced capacity for parasite transmission (22). TEP was identified via functional studies in *A. gambiae* mosquitoes as one of the key proteins important in the immunity of infection against *Plasmodium* (35). The expression of TEP1 in some strains of *A. gambiae* is responsible for the resistance of these mosquitoes to *Plasmodium* infections. Functional in vivo assays may be required to validate the role of TEP in *B. glabrata*, and provide a clearer picture of the role of this protein in the snail’s immune response.

The predicted protein-protein interaction network confirmed TEP binding to the APL1C/LRIM1 complex. Our prediction also shows that REL2 and an uncharacterized protein bind to the complex as presented in Fig. 1. The binding REL2 to AL1C may be vital in the activation and translational regulation of APL1C and the uncharacterized protein may play a role in the regulation of TEP. There is concrete evidence from both ‘wet’ and ‘dry’ laboratory experiment on the presence of TEP in insects like *Anopheles* mosquitoes (35) and *D. melanogaster,* and also investigated the role and mechanism of action of this protein in these arthropods (49).

Although, we employed *A. gambiae* model on the functional protein association networks since neither *B. glabrata* nor other Mollusca genome has not been included in the database. The organisms that are in the String database are those whose genome has been well annotated and published. Different crystal structures of *A. gambiae* TEP1 have been mapped and deposited in Protein Data Bank (http://www.rcsb.org/pdb/home/home.do). TEP had a highly reactive thioester motif, which can undergo spontaneous hydrolysis. This research identified these functional motifs as Alpha-macro-globulin thiol-ester bond-forming region. However, this vital motif was absent from *A. darling* (gb|ETN60497.1), *D. virilis* (gb|EDW64840.1) and *D. mojavensis* (gb|EDW12892.1) TEP homologues as presented in Fig. 3. If experimental research can validate the absent of thioester motif from these insects, it shows that they employ other proteins and mechanism in development of resistant to pathogens in strain. Alternatively, maybe not all strains of an insect express TEP as seen in *D. virilis* and *D. mojavensis*. One strain of *D. virilis* and *D. mojavensis* with the accession no gb|EDW65046.2, and gb|EDW11960.2 respectively have the TE motif while the same species with accession number gb|EDW64840.1 and gb|EDW12892.1 respectively lacks the TE motif.

## Conclusion

The presence of the TE motif in TEP-like protein and this motif is similar to that found in *A. gambiae*; therefore, this could open a new era of schistosomiasis control research by disrupting schistosome–snail compatibility. Moreover, our exploitation of the recently released *B. glabrata* genome for TEP was successful and an ongoing work on shotgun proteomic and bioinformatics analyses of the hemolymph of *B. glabrata* in our laboratory reveals some proteoforms of this protein. However, further experimental studies could involve sensitive genomic, transcriptomic and proteomics tools.
